# Titration-Based Assessment of Matrix Effects in Direct Infusion Mass Spectrometry

**DOI:** 10.1021/jasms.6c00220

**Published:** 2026-10-24

**Authors:** Pontus Persson, Gabor Toth, Natália Remper, Lucie Davidová, Ingela Lanekoff

**Affiliations:** † Department of Chemistry for Life Sciences, 8097 Uppsala University, 752 37 Uppsala, Sweden; ‡ Center of Excellence for the Chemical Mechanisms of Life, Uppsala University, 752 37 Uppsala, Sweden

## Abstract

Electrospray ionization mass spectrometry (ESI-MS) is a standard and robust technique for analysis of a large variety of biomolecules. Direct infusion approaches offer rapid, high-throughput analysis of small sample volumes but are inherently susceptible to matrix effects during electrospray ionization. These matrix effects can substantially alter analyte ionization and gas-phase response and thereby complicate the interpretation of analyte abundances in complex biological samples. Herein, we developed a titration-based workflow to identify matrix effects and distinguish true changes in endogenous analyte abundance from ionization artifacts using direct infusion probe mass spectrometry (DIP-MS). Rat brain tissue extracts, rat insulinoma cell extracts, and isotopically labeled internal standards were used to model different matrix complexities to define condition-based differences in matrix effects. Thereafter, by mixing paired extracts in different ratios and fitting linear regression models, we assessed analyte-specific responses to sample-specific matrices. Moreover, we uncovered ion suppression or enhancement of hundreds of analytes by comparing regression slopes using interaction tests. We find that using endogenous analytes in our titration-based workflow gives a good indication of matrix effects for certain analyte classes. However, using a preselected panel of internal standards is strongly favorable for reliably identifying matrix effects during ionization. Altogether, combining our titration workflow, a selected panel of internal standards, and regression modeling provides a sensitive way of detecting matrix effects and for distinguishing true metabolite abundance changes from ionization artifacts.

## Graphical abstract

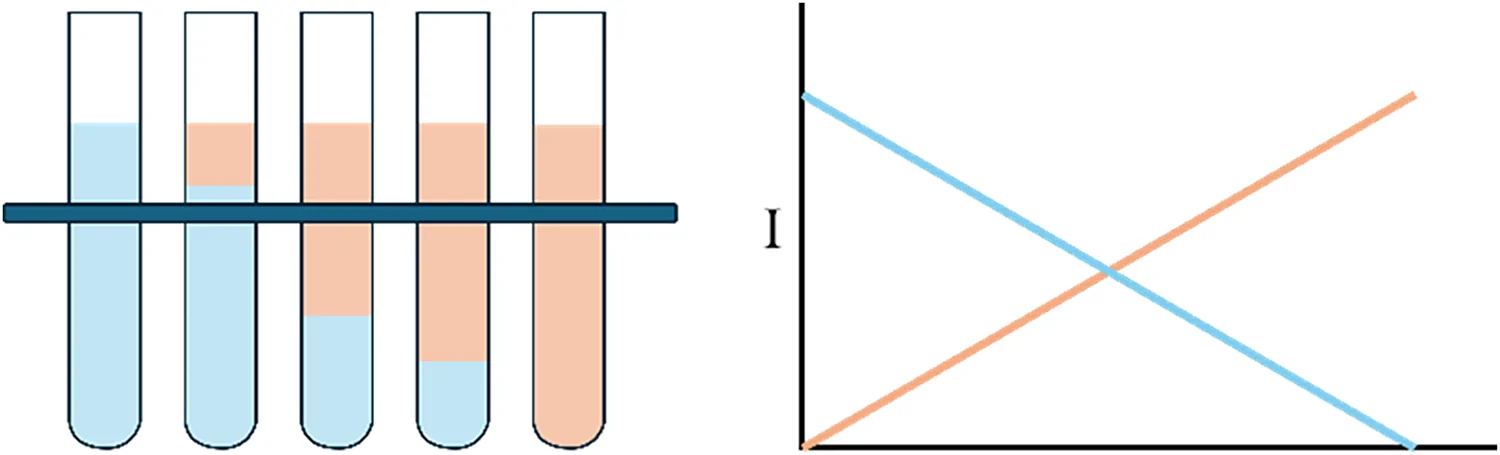

## Introduction

Mass spectrometry (MS) has become an important analytical tool for biomolecular analysis, offering high sensitivity and broad chemical coverage for small molecules without the need for labeling or targeting specific analytes.^1,2^ With chromatographic separation prior to MS, analytes can be separated prior to ionization for high-sensitivity and high-specificity analytical workflows. Approaches for direct infusion MS bypass chromatographic separation of individual analytes, enabling higher throughput.^3–8^ However, with direct infusion, as well as very rapid chromatography with coeluting analytes, the simultaneous ionization of all molecules in the matrix causes matrix effects to become extremely pronounced due to the high chemical complexity of the sample matrices. Major sources of matrix effects include salts, lipids, surfactants, metabolites, and solvent clusters.^9–11^ Additionally, high concentrations of standards and cross-contamination between samples may contribute to both ion suppression and enhancement. Although matrix effects occur in all ionization techniques, alterations to the chemical environment at the droplet surface are known to cause the most pronounced matrix effects in electrospray ionization (ESI), which may complicate data interpretation.^12–18^

In ESI, the matrix components directly influence ion formation and efficiency by competing both for charge and positions at the droplet surface.^19^ The extent of ion formation also depends on proton or cation affinity, molecular interactions among all species present in the interface, and depletion of available charge within droplets.^13,20,21^ Furthermore, nonvolatile matrix components may inhibit droplets from reaching their critical radius, and high concentrations of interfering compounds may increase droplet viscosity and surface tension, consequently reducing analyte transfer to the gas phase while promoting adduct or ion-pair formation.^22^ Reactions between analytes and nonvolatile species may also suppress analyte ionization and limit analyte transfer to the gas phase, and gas-phase reactions can contribute to the loss of analyte charge.^23,24^ Overall, matrix effects can substantially compromise analyte detection and quantification. As a result, they need to be mitigated or compensated for to correlate measured signal intensities to analyte abundance.^8,16^

Quantification in the presence of matrix effects in direct infusion typically relies on targeted analytes that are matched to appropriate internal standards. The selection of appropriate internal standards includes consideration of chemical class, adduct formation, and ionization mode.^25,26^ Mismatch between the internal standard and analyte can result in residual bias due to incomplete compensation for matrix effects.^27^ Therefore, isotopically labeled standards that have an almost identical ionization efficiency as the endogenous analytes are considered the golden standard. Consequently, fully quantitative workflows often focus on a defined panel of targets for which validated, labeled standards are available. However, this limits the use of the unique potential of mass spectrometry to perform nontargeted chemical analysis.^15,16^ In particular, discovery and screening applications require nontargeted coverage to detect unexpected or broad metabolic changes. In these approaches, it is not feasible to use internal standards or class-specific internal standards for all of the analytes. Therefore, there is an ongoing need to develop methodologies that can help overcome issues with matrix effects in nontargeted workflows, enabling quantitative comparisons across chemically complex sample matrices.

Here, we investigate a titration-based approach employing the direct infusion probe (DIP) for direct infusion MS to identify altered metabolite abundances in chemically complex samples. In this approach, two solutions are mixed in ratios of 0–100% and 100–0% with the aim of revealing both analyte-specific and global matrix-effect differences between the two solutions. Additionally, the solutions are doped with a restricted panel of internal standards to identify ion suppression or enhancement. Overall, we find that the approach enables more confident data interpretation for some metabolites, although internal standards are required for quantitative reliability of most of the investigated endogenous metabolites in tissue and cell extracts.

## Experimental Section

### Chemicals

Internal standards included C18:0 carnitine-d_3_ (stearoyl-l-carnitine-d_3_ (chloride)) (Larodan), ^15^N-labeled amino acid mix (Sigma-Aldrich) (Table S1), phosphatidylcholine (PC) 11:0/11:0 (1,2-diundecanoyl-*sn*-glycero-3-phosphocholine) (Avanti), lysophosphatidylcholine (LPC) 19:0 (1-nonadecanoyl-2-hydroxy-*sn*-glycero-3-phosphocholine) (Avanti), and monoacylglycerol (MG) 19:2 (10­(*Z*),13­(*Z*)-monononadecadienoin) (Larodan), and they were all prepared in 9:1 methanol:water (v/v) + 0.1% formic acid.

### Biological Samples

Fresh-frozen mouse brains were purchased from Creative Biolabs (NY, USA) and used to prepare a rat brain extract (RBE) containing 34.6 mg mL^–1^ of wet rat brain and stored. The extract was further diluted using 9:1 methanol:water (v/v) + 0.1% formic acid to the desired dilutions.^28^

Rat insulinoma cell line INS-1 clone 832/13 cells were maintained in RPMI 1640 (Invitrogen) containing 11.1 mM glucose and supplemented with 10% Newborn Calf Serum, l-glutamine (2 mM), penicillin (100 μg/mL), streptomycin (100 μg/mL), sodium pyruvate (1 mM), and β-mercaptoethanol (50 μM). Cells at 90% confluency were incubated for 1 h at 37 °C in an extracellular buffer (138 mM NaCl, 5.6 mM KCl, 1.2 mM MgCl_2_, and 2.6 mM CaCl_2_) with the addition of either low (L) glucose at 3 mM or high (H) glucose at 20 mM. Subsequently, the cells were lysed with a lysing solution of 9:1 methanol/water (v/v) + 0.1% formic acid doped with 2 μM LPC 19:0, diluted ^15^N-amino acid mix (Table S1), 0.5 μM C18:0 carnitine-d_3_, 2 μM MG 19:2, 5 μM PC 11:0/11:0, and 2.5 μM acetylcholine-d_9_. Both sets of cells contained 600,000 cells per mL. The cellular extracts were sonicated for 2 min, transferred into Eppendorf tubes, and centrifuged at 13,000 rpm for 5 min before the supernatants were collected.

### Samples

Four solutions (A1, A2, B1, and B2) contained both RBE and internal standards (Table 1). Titration samples were prepared by mixing A1 and B1 for experiment 1 and A2 and B2 for experiment 2 in the ratios of 10:0, 9:1, 7:3, 5:5, 3:7, 1:9, and 0:10 (Table S2). Samples for titration of the cellular extracts L and H were mixed in the ratios of 10:0, 9:1, 8:2, 7:3, 6:4, 5:5, 4:6, 3:7, 2:8, 1:9, and 0:10 (Table S1).

**Table 1:** Composition of Solutions Used for Titration Experiments to Study Matrix Effects^a^

Solution component	**A1**	**A2**	**B1**	**B2**
Rat brain extract	1:33 dilution	**1:17** **dilution**	1:33 dilution	**1:67** **dilution**
15N amino acid standard	1:2500 dilution	1:2500 dilution	1:2500 dilution	1:2500 dilution
LPC 19:0	5 μM	5 μM	5 μM	5 μM
**Carnitine** **C18:0-d_3_**	1 μM	1 μM		
**MG** 19:2	2 μM	2 μM		
**Acetylcholine-d_9_**			5 μM	5 μM
**PC** **11:0/11:0**			10 μM	10 μM

a Bold text emphasizes differences between the solutions.

### Mass Spectrometry

The direct infusion probe (DIP) was used for sample introduction and electrospray ionization.^8^ Measurements were conducted for 1 min on a Thermo LTQ Velos Pro Orbitrap (Thermo Fisher Scientific, Bremen, Germany) operated in positive ionization mode at a resolution of 100,000 (*m*/Δ*m* at *m*/*z* 400), capillary temperature of 300 °C, the S-Lens RF level was 69.4%, and the injection time was set to 300 ms. For experiments 1 and 2, the spray voltage was 2.40 kV, the N_2_ gas pressure for the DIP was 4000 mbar, and mass spectra were acquired in the range of *m*/*z* 85–1000. For experiment 3, the spray voltage was 2.30 kV, the N_2_ gas pressure for DIP was 3500 mbar, and the acquisition range *m*/*z* was 110–1000. All measurements were performed with a 1 min data acquisition time. Measurements of RBE samples were acquired in three technical replicates and cell extracts in five technical replicates.

### Data Analysis

Data from the DIP-MS measurements were converted to .mzML and extracted using an in-house developed MATLAB script. The data were further analyzed using Microsoft Excel by conducting linear regression analysis, multiple linear regression with interaction term analysis, and Student’s *t* tests. All statistical analyses were conducted with α = 0.05, and all *p*-values were FDR-corrected using Benjamini-Hochberg corrections.

## Results and Discussion

In DIP-MS, the sample is aspirated directly from the vial for subsequent electrospray ionization and MS analysis. Its design enables rapid analysis of metabolites and lipids from limited sample volumes in various analysis solvents.^8,18^ However, compared with chromatographic separation prior to MS, direct infusion techniques are sensitive to matrix effects caused by simultaneous infusion and ionization of thousands of molecules at the same time. With the rapid analysis enabled by DIP-MS, it provides an excellent opportunity to study matrix effects, including ion suppression and enhancement.

To evaluate matrix effects under ESI, chemically complex extracts were prepared from rat brain tissue (Figure 1A and Tables 1 and S2). Tissue was homogenized, and metabolites and lipids were extracted into methanol to produce a rat brain extract (RBE) as described in the method.^28^ Thereafter, RBE was diluted to vary the total concentration of molecules contributing to the chemical matrix. In one experiment, two solutions (A1 and B1), each containing equal amounts of RBE and a common set of standards (^15^N-labeled amino acids and LPC 19:0; Tables 1 and S2), were mixed in seven ratios in a titration series prior to DIP-MS analysis (Figure 1A). Specifically, solution A1 uniquely contained carnitine-d_3_ and MG 19:2, while B1 uniquely contained acetylcholine-d_9_ and PC 11:0/11:0 (Table 1). Thus, with the similarity of the two solutions, they were expected to have identical matrix effects and composition, except for the four differing internal standards.

**Figure 1:**
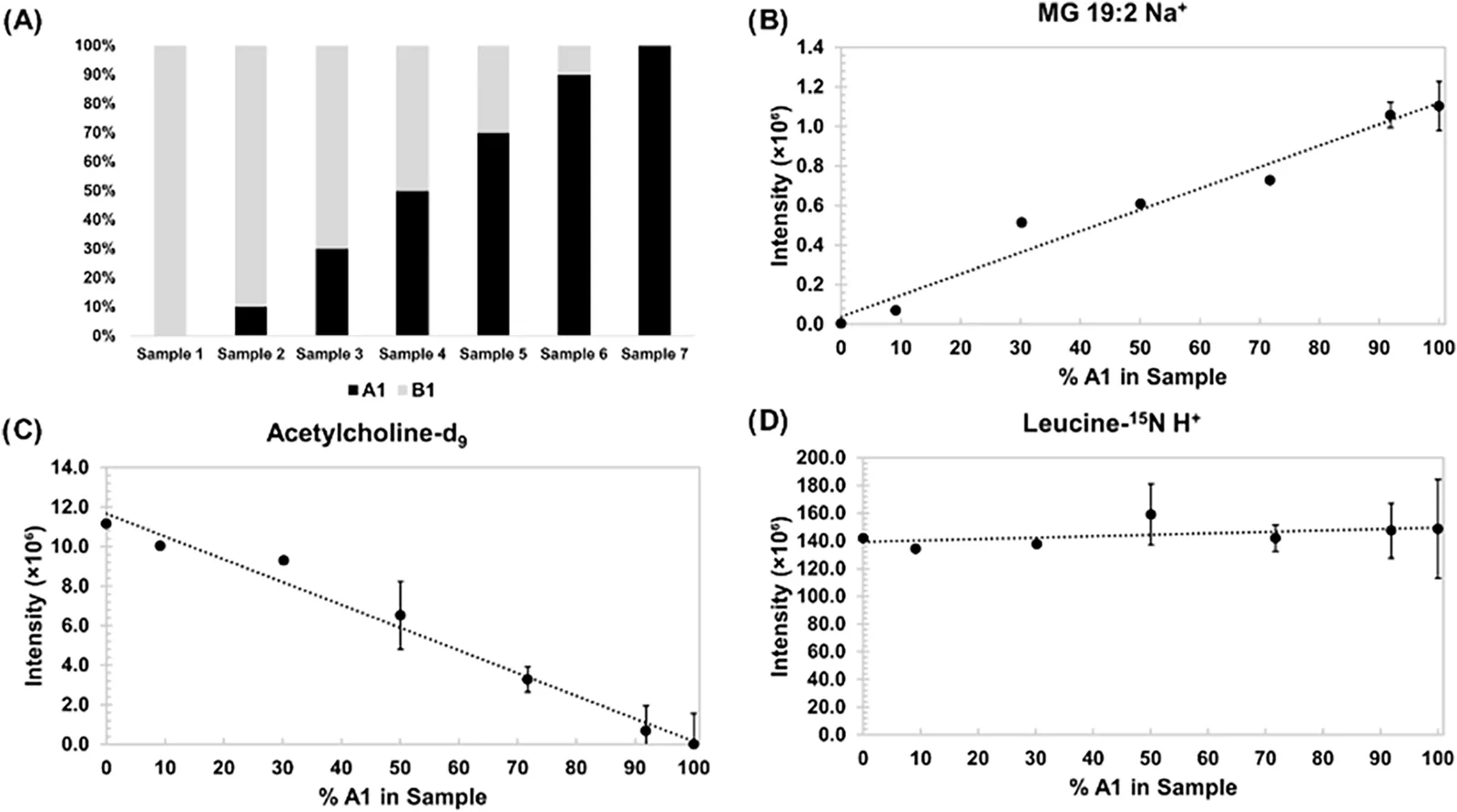
Titration of solutions A1 and B1, which have nearly identical chemical composition. (A) Schematic of the seven-point titration series generated by mixing A1 (black) and B1 (grey). (B) Signal intensity of [MG 19:2 + Na]^+^ (unique to A1). (C) Signal intensity of [acetylcholine-d_9_]^+^ (unique to B1); (D) Signal intensity of [^15^N-leucine + H]^+^ (common standard across seven samples). Error bars represent ±1 standard deviation of technical triplicate measurements. Data for slopes and *R* ^2^ are found in Table S3 and additional examples in Figure S1.

The seven titration samples were run with DIP-MS, and the data for selected standards were extracted to visualize the experimental results as a proof of concept. It was found that as the proportion of A1 increased, the signal intensity of MG 19:2 increased and that of acetylcholine-d9 decreased (Figure 1B,C), confirming the expected behavior of the unique internal standards. By contrast, the common internal standard ^15^N-leucine and other labeled amino acids showed constant intensities with moderate standard deviations (<11% for ^15^N-leucine) across the titration (Figures 1D and S1). The consistent internal standard responses demonstrate that the similar compositions of A1 and B1 did not yield measurable matrix-effect differences.

When analyzing biological samples, it is almost impossible to generate two identical chemical compositions that ensure that the analysis is free of matrix effects. To assess how gradually increasing/decreasing chemical matrix concentration affects ionization, the same titration approach was used in Experiment 2. An exaggerated model system was constructed where solution A2 was prepared with doubled RBE concentration (2 × RBE) while B2 had a halved RBE concentration (0.5 × RBE) (Table 1). Thus, the total chemical concentration was 4 times higher in A2 compared to B2. Following, seven titration samples were mixed from A2 and B2 in the same way as in Experiment 1 (Figure 1A). Consequently, the samples had an increasing proportion of A2 and therefore increasing overall chemical concentration, which is expected to increase matrix effects.

The data from Experiment 1 with constant matrix (black) and Experiment 2 with increased matrix concentration (orange) are plotted together for direct comparison (Figure 2). The results show that the intensity of [LPC 19:0 + H]^+^, which was added at the same concentration (5 μM) in both A2 and B2 and remained constant across the titration in Experiment 1 (constant matrix, black), decreased significantly (*p* < 0.001, regression comparison including an interaction term) as the A2 solution was increased in the samples (Figure 2A, orange). This means that the signal of [LPC 19:0 + H]^+^ decreases as the chemical matrix concentration increases, despite the constant concentration. We therefore conclude that it is subject to ion suppression. A similar but smaller suppression was observed for [^15^N-leucine + H]^+^, which was also present at equal concentration in A2 and B2 (*p* = 0.005, Figure 2B). These differences in experienced ion suppression between LPC and leucine suggest that their structural differences position them in different places in the droplet.^29^

**Figure 2:**
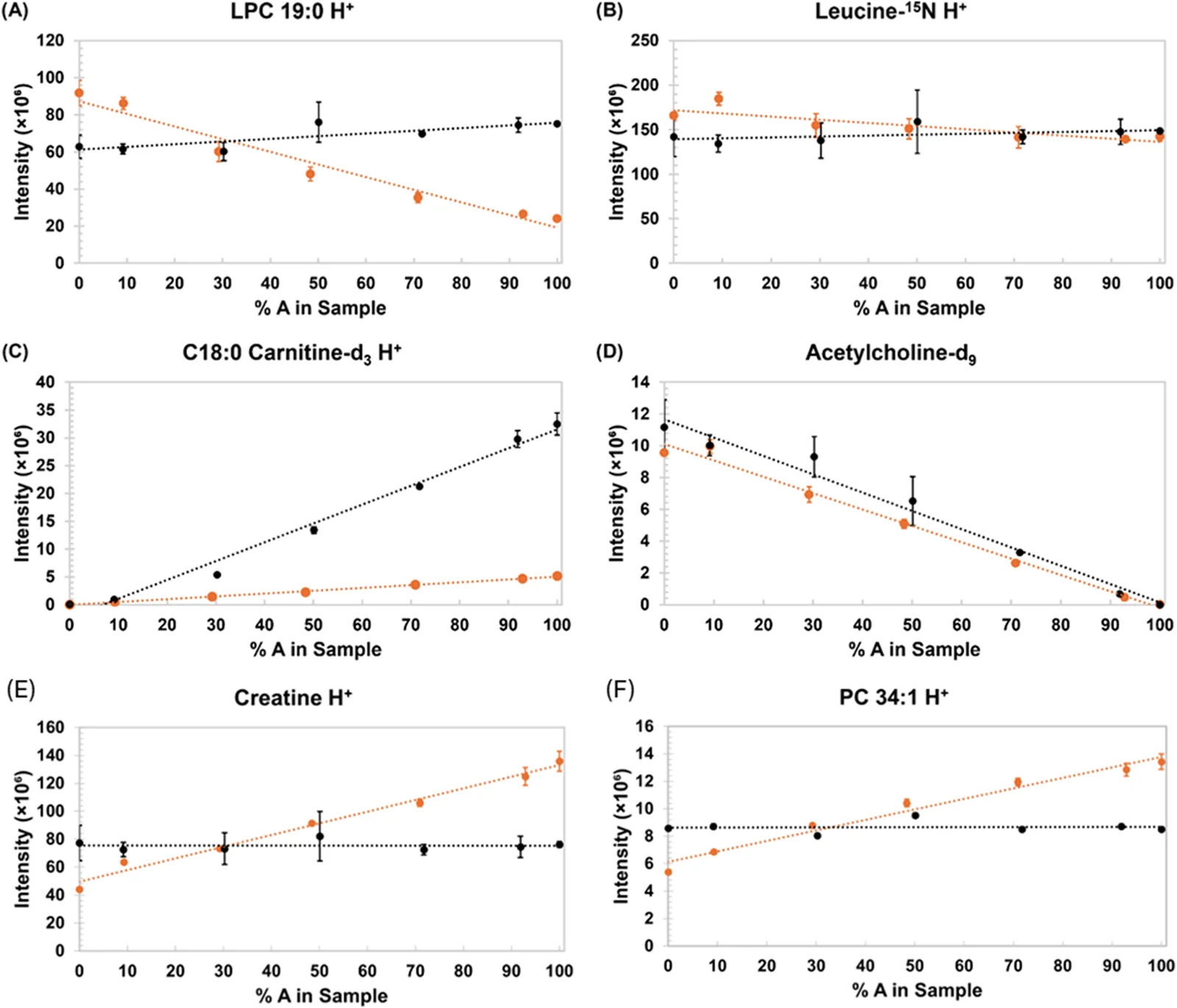
Comparison of experimentally obtained slopes from experiment 1 (constant RBE, black) and experiment 2 (increased RBE complexity, orange). Data from both experiments are plotted together for direct comparison of linear responses across the 14 titration samples with increased ratios of A1 or A2 solutions in the samples. (A) [LPC 19:0 + H]^+^; (B) [^15^N-leucine + H]^+^; (C) [Carnitine C18:0-d_3_ + H]^+^ (unique to A); (D) [acetylcholine-d_9_]^+^ (unique to B); (E) [creatine + H]^+^ (endogenous in RBE); (F) [PC 34:1 + H]^+^ (endogenous in RBE). Error bars represent ±1 standard deviation of technical triplicate measurements. Table S3 includes slopes, *R* ^2^, and reported *p*-values that refer to the interaction test comparing the two linear regressions (difference in slopes), and additional examples are shown in Figures S2 and S3.

When comparing the two experiments, it becomes clear that standards that are unique to one solution also showed altered responses as the matrix complexity increased. For example, [Carnitine C18:0-d_3_ + H]^+^ (1 μM, unique to A) increased in intensity as expected with a higher A2 fraction in experiment 2. However, its slope was significantly (*p* < 0.001) reduced compared to experiment 1, where the matrix was constant, which implies the effect of ion suppression (Figure 2C). By contrast, acetylcholine-d_9_ (5 μM, unique to B) exhibited similar slopes in both experiments, suggesting that it does not experience ion suppression (*p* = 0.191). This resistance to ion suppression is likely an effect of the molecule’s permanent positive charge, making it less affected by the competition for charge during ionization (Figure 2D).

In addition to the common and unique internal standards added to the A2 and B2 solutions, the RBE contains endogenous molecules. While the majority of endogenous analytes were not annotated, it was clear that, as the concentration of RBE increases with increased A2 fraction, the intensity of the endogenous molecules increases. This is supported by the tentatively annotated [creatine + H]^+^ and [PC 34:1 + H]^+^ titration curve plots that both show a significant (*p* < 0.001 for both) increase compared to when the RBE was constant (Figure 2E,F). Important considerations include the ionization efficiency, analyte physicochemical properties, and cation affinity for positive ionization mode.^19,29^

These results demonstrate that the titration approach effectively reveals analyte-specific matrix effects in chemically complex samples through the use of internal standards. This is attributed to two features of the method: (i) the titration curves provide intuitive visualizations, (ii) an increased number of samples analyzed contributes to better sensitivity in detecting subtle differences due to increased statistical power. While performing titration series in general means extra effort in hands-on work, the increase in analysis time is negligible due to the high-throughput nature of the DIP-MS technique. Nevertheless, DIP titrations provide a fast and easy way of assessing matrix effects for subsequent experimental planning.

To model a more realistic scenario, Experiment 3 was conducted to compare cellular extracts from INS-1 cells exposed to low (L, 3 mM) or high (H, 20 mM) glucose for one hour. High-glucose treatment is known to alter INS-1 intracellular metabolism, increasing ATP and Ca^2+^ influx and triggering insulin release; therefore, an altered metabolome in the high-glucose condition is expected.^30^ However, whether the metabolic changes upon this glucose exposure would substantially modify the sample matrix and thereby cause ion suppression or enhancement is not known. For Experiment 3, methanolic extracts were prepared from 600,000 cells/mL per condition (L or H) and spiked with equal concentrations of a limited panel of internal standards (Table S3). Following this, samples for an 11-point titration series in 10% increments of the L and H extracts (0–100%) were generated, producing samples with increasing volumetric contribution of H extract (Figure S4). If the cellular extracts are chemically equal, the metabolites that are altered upon high-glucose treatment should be easily identified by the titration approach, since their linear regression slopes would be positive or negative, while indifferent metabolite abundances are reflected by constant lines. For the results, both the raw intensities of tentatively annotated endogenous metabolites in dark green diamonds and corresponding internal standards in green squares are displayed for comparison (Figures 3 and S5).

**Figure 3:**
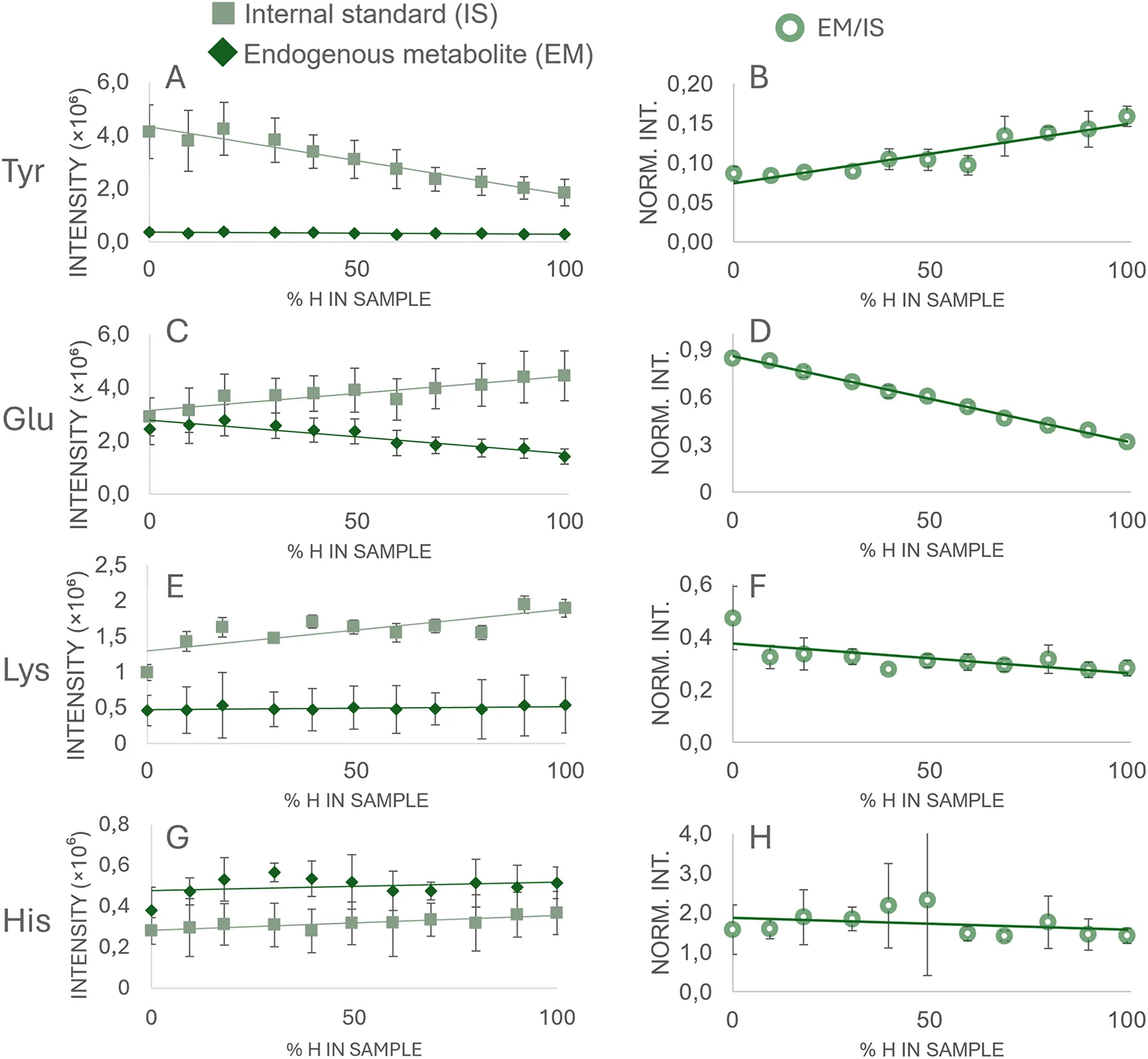
Titration approach combined with internal standards to assess matrix effects and identify altered metabolites in INS-1 cell extracts across 11 titration samples with increasing fraction of H extract (0–100%). Endogenous metabolites are shown with dark green diamonds and the ^15^N-labeled internal standards with green squares. (A) Intensities of endogenous [tyrosine + H]^+^ and internal standard [^15^N-tyrosine + H]^+^. (B) Ratio [tyrosine + H]^+^ to [^15^N-tyrosine + H]^+^; (C) [glutamate + H]^+^ and [^15^N-glutamate + H]^+^; (D) Ratio [glutamate + H]^+^ to [^15^N-glutamate + H]^+^; (E) [lysine + H]^+^ and [^15^N-lysine + H]^+^; (F) Ratio [lysine + H]^+^ to [^15^N-lysine + H]^+^; (G) [histidine + Na]^+^ and [^15^N-histidine + Na]^+^; (H) Ratio [histidine + Na]^+^ to [^15^N-histidine + Na]^+^. H = cellular extract from high-glucose (20 mM) treated INS-1 cells spiked with internal standards; L = cellular extract from low-glucose (3 mM) treated INS-1 cells spiked with internal standards. Error bars show ±1 standard deviation of five technical replicates. Data for linear regression slopes, *R* ^2^ values, *p*-values from the multiple linear regression including an interaction term (test for difference in slopes between IS and EM), and *p*′ = *p*-value for the linear regression shown on normalized titration plots are found in Table S4.

The linear regression for endogenous [tyrosine + H]^+^ shows a flat response across the titration samples, indicating that tyrosine is not affected by high-glucose treatment (Figure 3A). However, the slope for the internal standard [^15^N-tyrosine + H]^+^, added at equal concentration to both extracts, is significantly different (*p* < 0.001) and decreased in intensity as the fraction of H rose. This suggests that there is an effect of ion suppression of the tyrosine internal standard in the H sample. Normalizing the intensity from endogenous tyrosine to the internal standard, and thereby removing matrix effects,^12,31,32^ shows that endogenous tyrosine is increased in cells exposed to high glucose (Figures 3B and S6). Almost opposite results are found for glutamate, where the slope of endogenous signal [glutamate + H]^+^ decreases as the H fraction increases (Figure 3C). Contrarily, the slope of the corresponding internal standard [^15^N-glutamate + H]^+^ is significantly (*p* < 0.001) different, with an increasing slope, suggesting ion enhancement as H increases in the titration samples. Despite the increase in endogenous glutamate signal, normalization to the internal standard shows that glutamate is significantly (*p*′ < 0.001) decreased in H cell extracts (Figure 3D).

For endogenous [lysine + H]^+^, the intensity remained essentially unchanged across the titration, while the slope of the internal standard [^15^N-lysine + H]^+^ increased with higher proportions of H extract (*p* < 0.001) (Figure 3E). After normalization, the slope shows a significant decrease in lysine in high-glucose-treated cells (*p*′ < 0.001). Importantly, in this case, the titration approach offers higher confidence for the decrease (*p*′ < 0.001) compared to only analyzing 100% L and 100% H (*p*″ = 0.039) (Figure S6). Finally, results for histidine suggest that it is not affected by matrix effects and that it is not altered in the high-glucose-treated cells (*p* = 0.651, *p*′ = 0.349) (Figure 3G,H and Table S4). Overall, the results show that tyrosine, glutamate, and lysine are altered in cells exposed to high glucose. Furthermore, tyrosine, glutamate, and lysine were subjected to matrix effects, including ion suppression and enhancement, caused by the different chemical matrices of cellular extracts from cells treated with low or high glucose.

The above results show that our titration-based DIP-MS strategy is useful for the intuitive assessment of matrix effects and for relative quantitation of endogenous analytes. Although adding a small panel of internal standards in equal concentrations is necessary to account for matrix effects even when titrating two solutions, the titration strategy, while slightly increasing the required effort, offers clear benefits over running the extracts one-by-one. Plotting internal standard-normalized endogenous signals with linear regressions provides a clear visualization of concentration changes for endogenous compounds (Figure 3). Furthermore, the normalized regression plots give stronger and more informative evidence than pairwise *t* tests alone, as demonstrated for lysine (*p*′ < 0.001 and *p*″ = 0.039). Overall, the titration approach is useful for assessing matrix effects and comparing metabolite alterations in two chemically complex solutions, but it still requires the use of proper internal standards.

## Conclusion

We presented a platform combining a rapid DIP-MS workflow with a titration-based strategy to provide an unbiased approach for evaluating matrix effects across chemically complex samples. Although it would be a much larger time investment, a similar approach could be used to investigate matrix effects in LC-MS. Mixing two extracts across a 0–100% series and including a limited panel of internal standards enabled direct detection of matrix-dependent ion suppression or enhancement while simultaneously identifying true changes in endogenous analyte abundances. In particular, comparing regression slopes of different analytes using interaction tests for condition comparisons provides a sensitive, independent metric that outperforms single-sample comparisons in certain cases. We have leveraged the developed approach to decipher analyte-specific ionization behavior of small metabolites in both tissue and cell extracts (e.g., leucine and acetylcholine). We find that matrix effects also exist when using the same cell culture but different treatments. Overall, combining direct infusion DIP-MS with a titration strategy and internal standard normalization provides a broadly applicable platform for metabolomics and lipidomics studies requiring robust, high-throughput screening of large sample sets.

## Supplementary Material



## Notes

Concentrations of 15N-labeled amino acids (Table S1); composition of samples for experiments 1–3 (Table S2); data from Experiment 1 and Experiment 2 (Table S3); plots of data from Experiment 1 (Figure S1); plots of data from Experiments 1 and 2 (Figure S2); plots of data from Experiments 1 and 2 (Figure S3); samples for Experiment 3 (Figure S4); plots of data from Experiment 3 (Figure S5); and plots of data from Experiment 3 (Figure S6) (PDF)

## §

Department of Clinical Pharmacology, Laboratory of Drugs of Abuse Analysis, Karolinska University Hospital, Stockholm, Sweden

## Abbreviations

DIP: direct infusion probe
IS: internal standard
LPC: lysophosphatidylcholine
MS: mass spectrometry
PC: phosphatidylcholine
RBE: rat brain extract
