# Supplementary material for: Titration-Based Assessment of Matrix Effects in Direct Infusion Mass Spectrometry
Source: J Am Soc Mass Spectrom. 2026 Oct 24;37(11):2742–8. doi: 10.1021/jasms.6c00220 (PMC13741900; doi:10.1021/jasms.6c00220)
Supplement: Supplementary file 1 [file js6c00220_si_001.pdf]

SUPPORTING INFORMATION

Titration-Based Assessment of Matrix Effects in Direct Infusion Mass Spectrometry

Pontus Persson[1], Gabor Toth[1,2†], Natália Remper[1], Lucie Davidová[1] and Ingela Lanekoff*[1,2]

[1]Department of Chemistry for Life Sciences, Uppsala University, 752 37 Uppsala, Sweden
[2]Center of Excellence for the Chemical Mechanisms of Life, Uppsala University, 752 37 Uppsala, Sweden

*Corresponding author
Prof. Ingela Lanekoff,
Department of Chemistry for Life Sciences,
Husargatan 3, 75 123 UPPSALA, Sweden
Email: ingela.lanekoff@kemi.uu.se

Table S1. Concentrations of 15N labelled amino acids in standard mixture and solution.

15N-labelled amino acid	Standard mixture concentration (µM)	Concentration in A1, A2, B1, B2 (µM)	Concentration in cell experiment (µM)
ASP	600	24	16
THR	350	14	9.33
SER	350	14	9.33
GLU	400	16	10.67
PRO	200	8	5.33
GLY	1000	40	26.67
ALA	1000	40	26.67
VAL	400	16	10.67
MET	100	4	2.67
ILE	300	12	8
LEU	450	18	12
TYR	100	4	2.67
PHE	160	6.4	4.26
HIS	50	2	1.33
LYS	150	6	4
ARG	100	4	2.67
GLN	200	8	5.33
ASN	200	8	5.33
TRP	200	8	5.33
CYS	200	8	5.33

Table S2. Composition of samples for titration approach in experiments 1-3.

Experiment 1		Experiment 2		Experiment 3	
A1	B1	A2	B2	L	H
10	0	10	0	10	0
9	1	9	1	9	1
7	3	7	3	8	2
5	5	5	5	7	3
3	7	3	7	6	4
1	9	1	9	5	5
0	10	0	10	4	6
				3	7
				2	8
				1	9
				0	10

Table S3. Data from Experiment 1 and Experiment 2. All p-values are FDR corrected using Benjamini-Hochberg correction.

	Black<br>Exp 1 - Constant RBE		Orange<br>Exp. 2 - Varying RBE		
	slope	R2	slope	R2	p
LPC 19:0	1.4E+05	0.67	-6.8E+05	0.96	<0.001
15N Leucine	1.0E+05	0.25	-3.5E+05	0.74	0.005
C18:0 Carnitine-d3	3.4E+05	0.98	-5.1E+04	0.998	<0.001
Acetylcholine-d9	-1.1E+05	0.98	-1.0E+05	0.99	0.19
Creatine	-4.7E+01	0	8.4E+05	0.99	<0.001
MG 19:2	1.1E+04	0.97	6.5E+03	0.99	<0.001
PC 34:1	6.4E+02	0	7.7E+04	0.97	<0.001

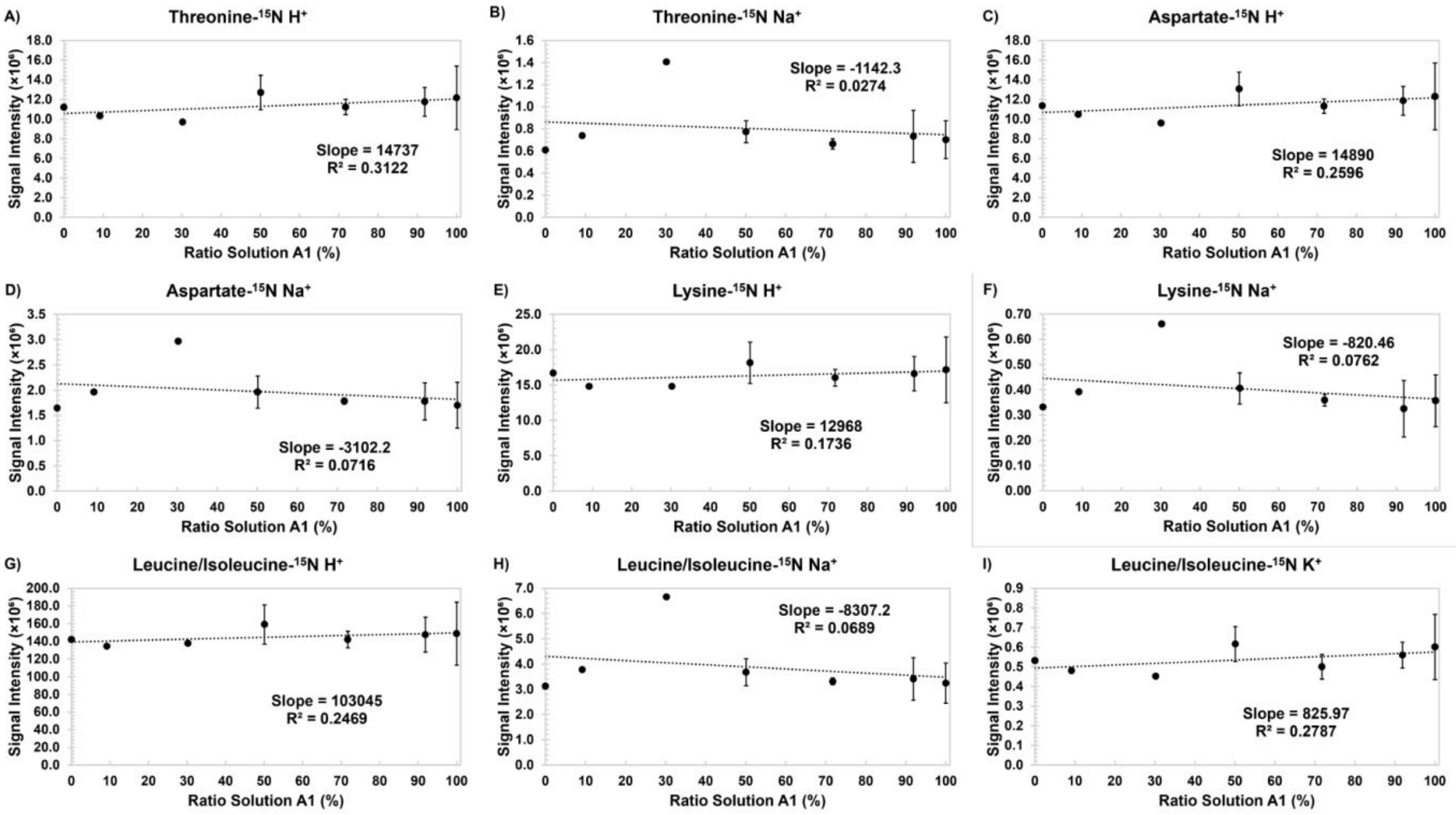

Figure S1. Intensity of selected 15N labelled amino acids when the chemical matrix was almost identical in solutions A1 and B1. The following ions of the internal standards are depicted: A) [Threonine-^{15}N+H]$^+$, B) [Threonine-^{15}N+Na]$^+$, C) [Aspartate-^{15}N+H]$^+$, D) [Aspertate-^{15}N+Na]$^+$, E) [Lysine-^{15}N+H]$^+$, F) [Lysine-^{15}N+Na]$^+$, G) [Leucine/Isoleucine-^{15}N+H]$^+$, H) [Leucine/Isoleucine-^{15}N+Na]$^+$, I) [Leucine/Isoleucine-^{15}N+K]$^+$. Error bars show one standard deviation and the slopes and R2 are displayed in the individual graphs.

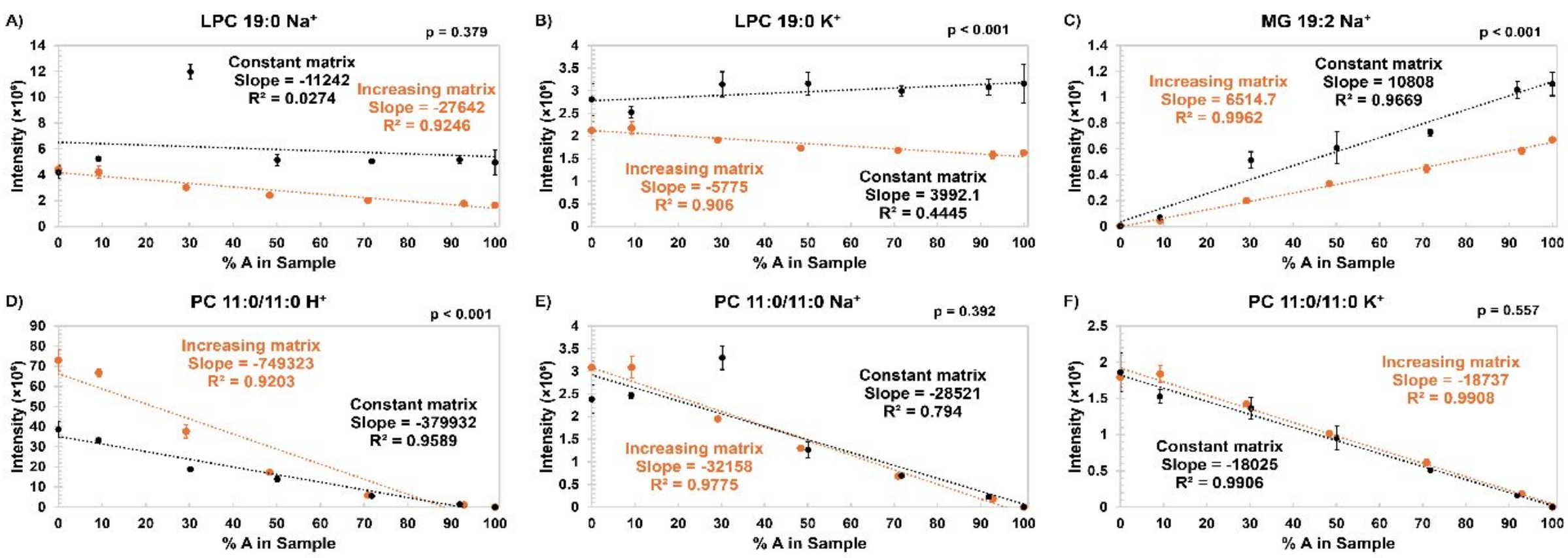

Figure S2. Intensity of selected lipid standards when the chemical matrix was increased. The black slope shows data from experiment 1, when the chemical matrix was almost identical, and the orange slope shows data from experiment 2, where the chemical matrix was increased in the samples due to A2 having 2xRBE whilst B2 had 0.5xRBE. The following ions of the internal standards are depicted: A) [LPC 19:0+Na]$^+$, B) [LPC 19:0+K]$^+$, C) [MG 19:2+Na]$^+$, D) [PC 11:0/11:0+H]$^+$, E) [PC 11:0/11:0+Na]$^+$, F) [PC 11:0/11:0+K]$^+$. Error bars show one standard deviation and the slopes and R2 are displayed in the individual graphs with color codes as described above. p-values from the multiple linear regression including an interaction term (test for difference in slopes between Exp. 1 and Exp. 2). All p-values are FDR corrected using Benjamini-Hochberg correction.

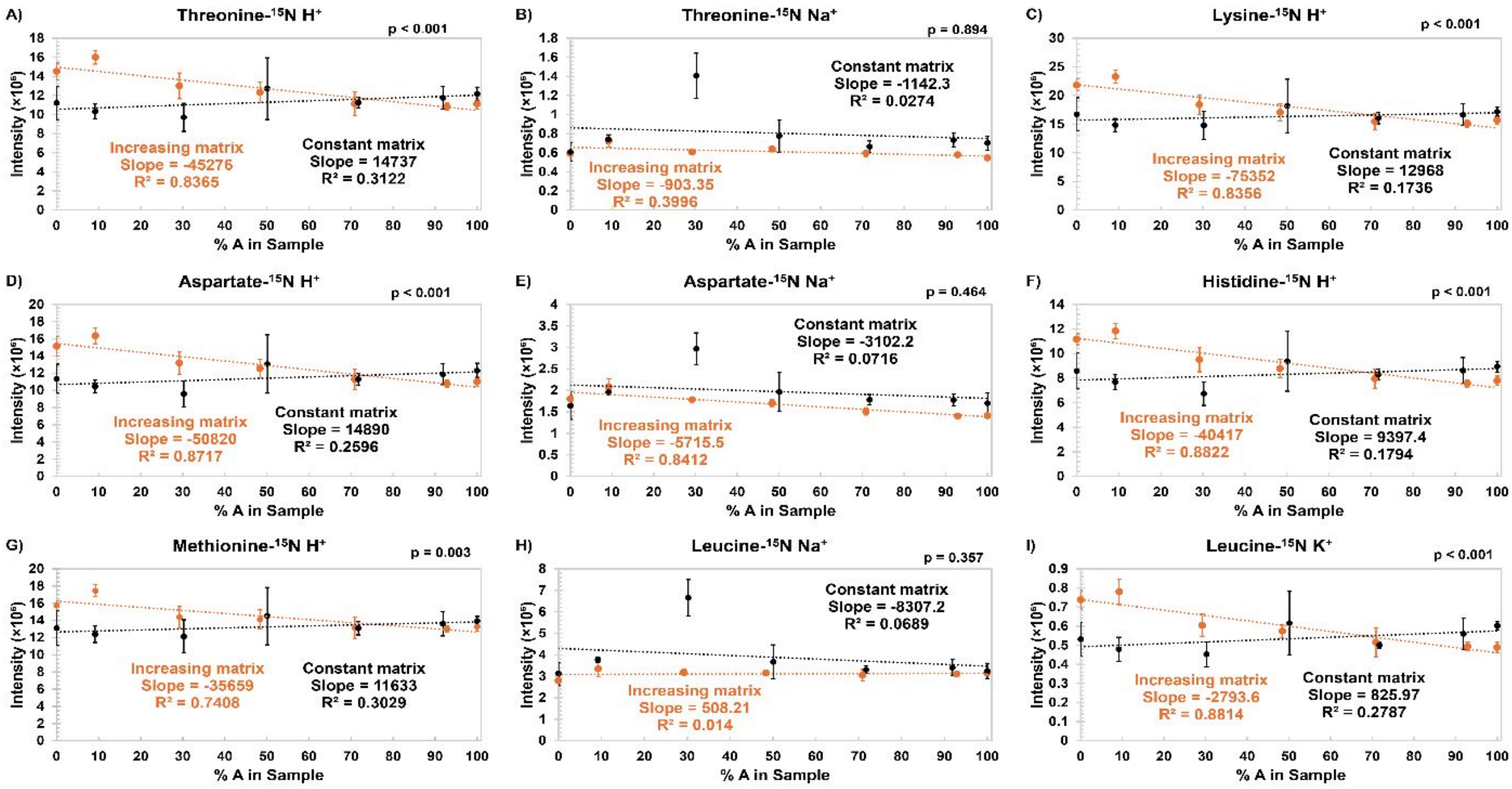

Figure S3. Intensity of selected 15N labelled amino acid internal standards (IS) when the chemical matrix was increased. The black slope shows data from experiment 1, when the chemical matrix was almost identical, and the orange slope shows data from experiment 2, where the chemical matrix was increased in the samples due to A2 having 2xRBE whilst B2 had 0.5xRBE. The following ions of the internal standards are depicted: A) [Threonine-^{15}N+H]$^+$, B) [Threonine-^{15}N+Na]$^+$, C) [Lysine-^{15}N+H]$^+$, D) [Aspartate-^{15}N+H]$^+$, E) [Aspertate-^{15}N+Na]$^+$, F) [Histidine-^{15}N+H]$^+$, G) [Methionine-^{15}N+H]$^+$, H) [Leucine/Isoleucine-^{15}N+Na]$^+$, I) [Leucine/Isoleucine-^{15}N+K]$^+$. Error bars show one standard deviation and the slopes and R2 are displayed in the individual graphs. p-values from the multiple linear regression including an interaction term (test for difference in slopes between Exp. 1 and Exp. 2). All p-values are FDR corrected using Benjamini-Hochberg correction.

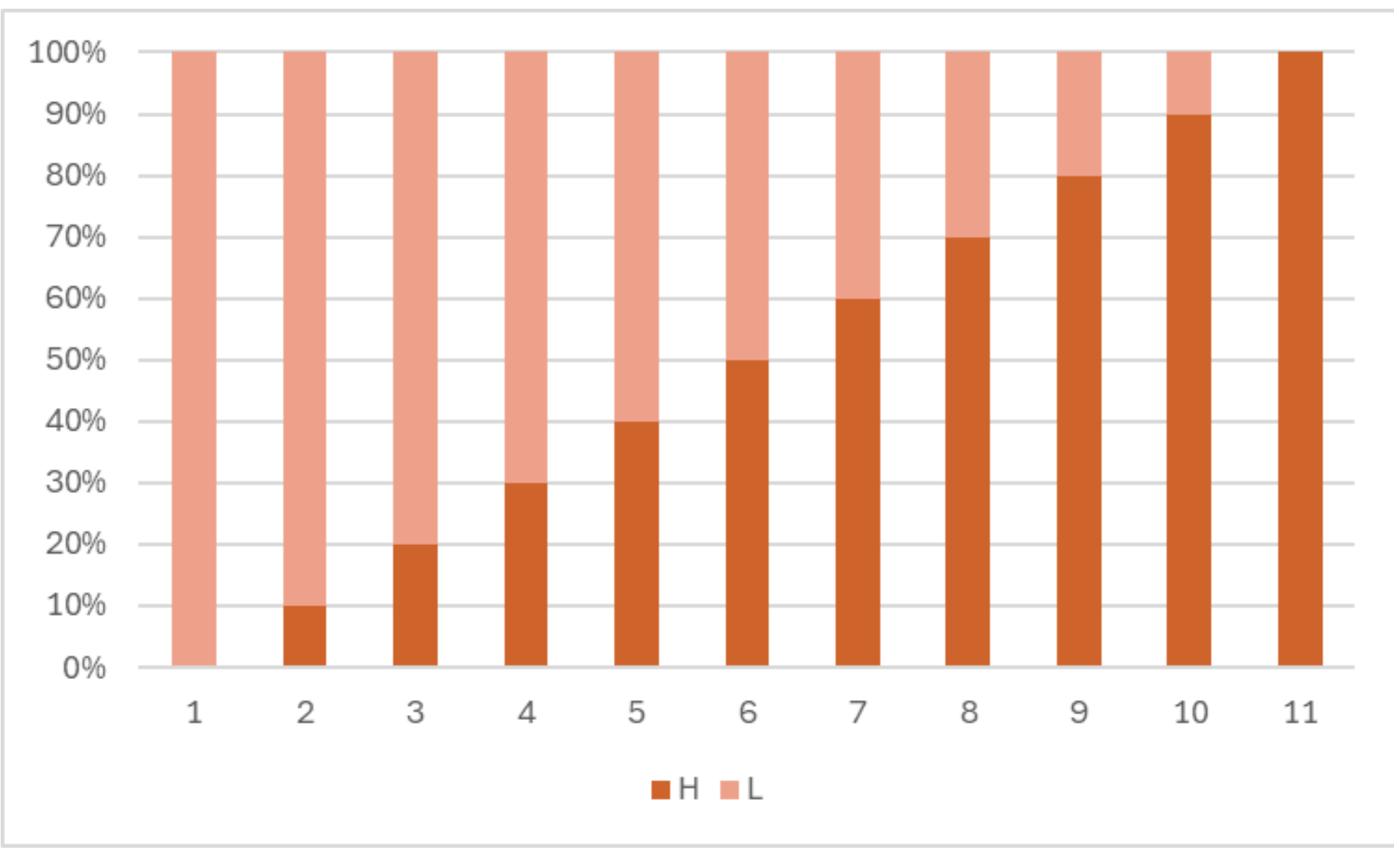

Figure S4. Titration samples for cellular extracts of INS-1 cells exposed to low (L) or high (H) glucose.

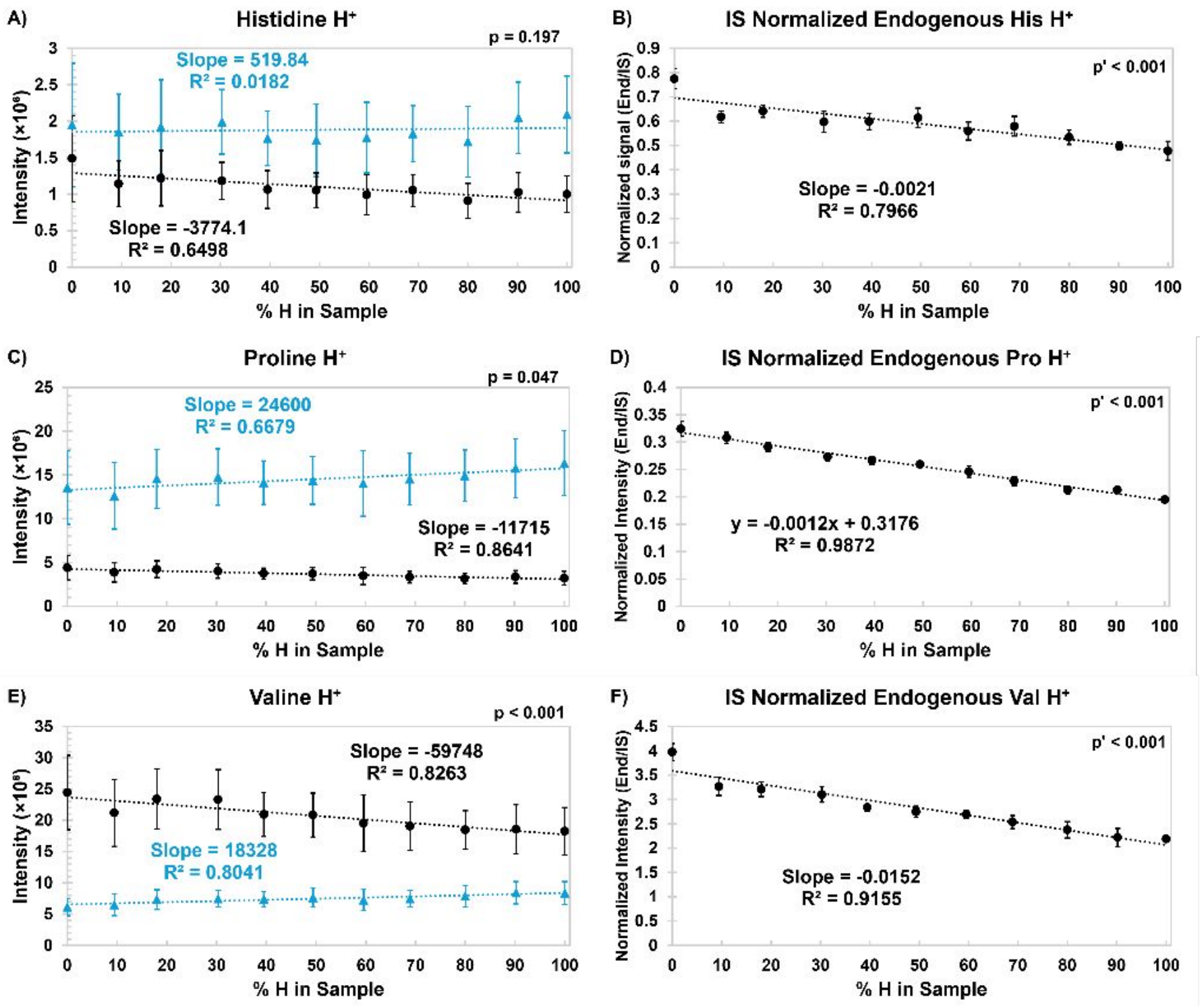

Figure S5. Additional examples of results from experiment 3. The following ions of the internal standards (IS) (blue) and endogenous metabolites (EM) (black) are depicted in A, C, and E and the endogenous ion normalized to the internal standard in B), D), and F): A) [Histidine-^{15}N+H]$^+$ and [Histidine+H]$^+$, B) [Histidine+H]$^+$ normalized to [Histidine-^{15}N+H]$^+$ C) [Proline-^{15}N+H]$^+$ and [Proline+H]$^+$, D) [Proline+H]$^+$ normalized to [Proline-^{15}N+H]$^+$ E) [Valine-^{15}N+H]$^+$ and [Valine+H]$^+$, F) [Valine+H]$^+$ normalized to [Valine-^{15}N+H]$^+$. Error bars show one standard deviation and the slopes and R2 are displayed in the individual graphs. p-values from the multiple linear regression including an interaction term (test for difference in slopes between IS and EM), and p′ = p-value for the linear regression shown on normalized titration plots. All p-values are FDR corrected using Benjamini-Hochberg correction.

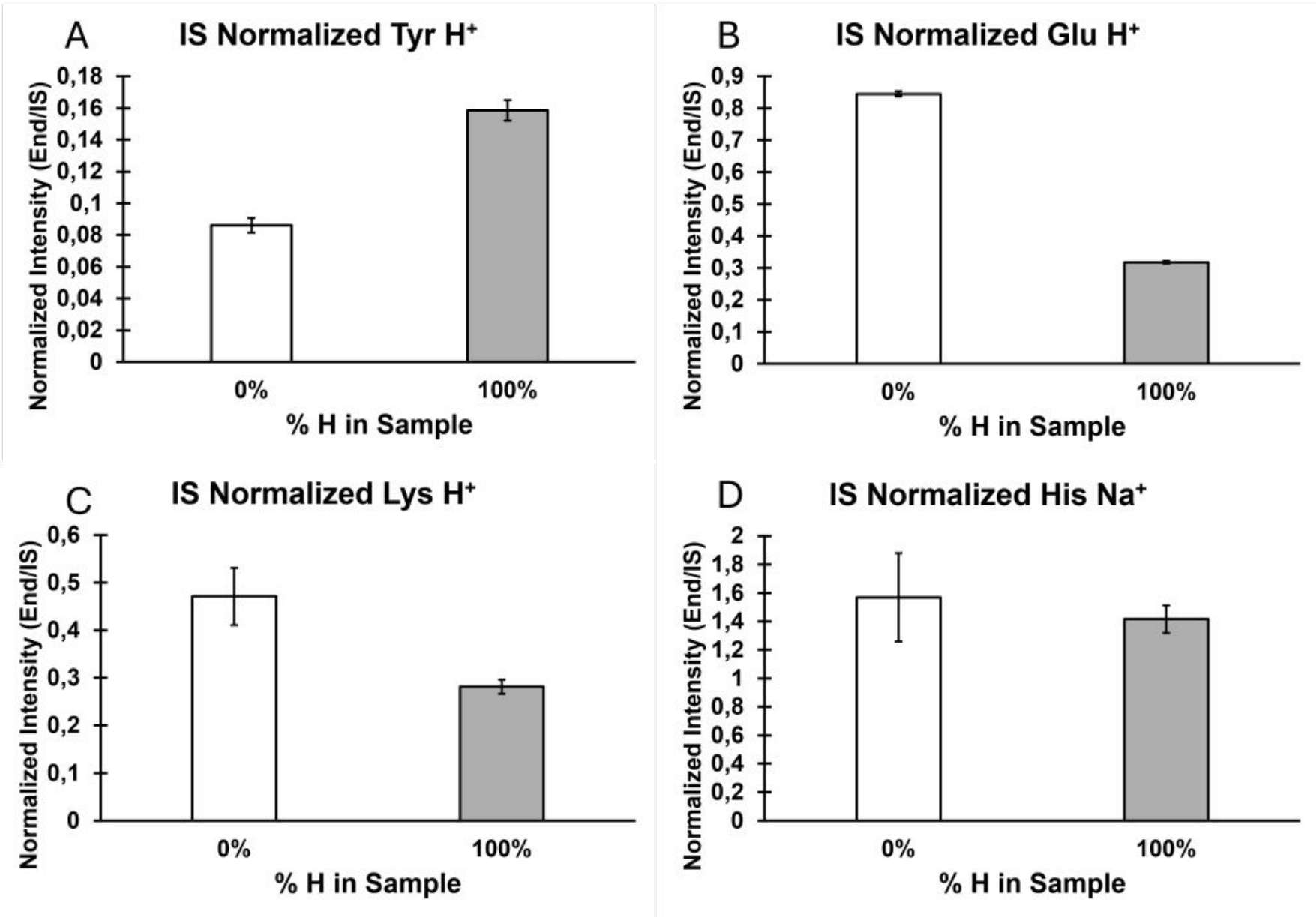

Figure S6. Comparison of normalized intensities of the endogenous metabolite to the corresponding internal standard (IS) for 100% L (0% H) and 100% H samples. The following ions are depicted: A) $[\text{Tyrosine+H}]^+$ normalized to $[\text{Tyrosine-}^{15}\text{N+H}]^+$, p’’<0.001, B) $[\text{Glutamate+H}]^+$ normalized to $[\text{Glutamate-}^{15}\text{N+H}]^+$, p’’<0.001 C) $[\text{Lysine+H}]^+$ normalized to $[\text{Lysine-}^{15}\text{N+H}]^+$, p’’=0.039 D) $[\text{Histidine+Na}]^+$ normalized to $[\text{Histidine-}^{15}\text{N+Na}]^+$, p’’=0.658. All p-values are FDR corrected using Benjamini-Hochberg correction.

Table S4. Data for linear regression slopes, R2 values and p-values for Figure 3. All p-values are FDR corrected using Benjamini-Hochberg correction.

	slope	R2	p	p'	p''
Tyrosine H^+	-737	0.52		-	-
15N-Tyrosine H^+	-25451	0.95	<0.001	-	-
Tyr/15N-Tyr H^+	0	0.87	-	<0.001	<0.001
Glutamate H^+	-12582	0.86		-	-
15N-Glutamate H^+	12895	0.83	<0.001	-	-
Glu/15N-Glu H^+	0	0.99	-	<0.001	<0.001
Lysine H^+	443	0.27		-	-
15N-Lysine H^+	5863	0.6	0.006	-	-
Lys/15N-Lys H^+	0	0.46	-	<0.001	0.039
Histidine Na^+	418	0.08		-	-
15N-Histidine Na^+	723	0.74	0.806	-	-
His/15N-His Na^+	0	0.1	-	0.349	0.658
